# A Shift from Cellular to Humoral Responses Contributes to Innate Immune Memory in the Vector Snail *Biomphalaria glabrata*

**DOI:** 10.1371/journal.ppat.1005361

**Published:** 2016-01-06

**Authors:** Silvain Pinaud, Julien Portela, David Duval, Fanny C. Nowacki, Marie-Aude Olive, Jean-François Allienne, Richard Galinier, Nolwenn M. Dheilly, Sylvie Kieffer-Jaquinod, Guillaume Mitta, André Théron, Benjamin Gourbal

**Affiliations:** 1 University of Perpignan Via Domitia, IHPE UMR 5244, CNRS, IFREMER, University of Montpellier, Perpignan, France; 2 Institute of Biological, Environmental & Rural Sciences, Aberystwyth University, Aberystwyth Ceredigion, United Kingdom; 3 School of Marine and Atmospheric Sciences, Stony Brook University, Stony Brook, New York, United States of America; 4 Plate-forme d'analyses protéomiques EDyP-Service, Laboratoire de Biologie à Grande Echelle UMR-S 1038 Inserm/CEA/UJF CEA, Grenoble, France; George Washington University School of Medicine and Health Sciences, UNITED STATES

## Abstract

Discoveries made over the past ten years have provided evidence that invertebrate antiparasitic responses may be primed in a sustainable manner, leading to the failure of a secondary encounter with the same pathogen. This phenomenon called “immune priming” or "innate immune memory" was mainly phenomenological. The demonstration of this process remains to be obtained and the underlying mechanisms remain to be discovered and exhaustively tested with rigorous functional and molecular methods, to eliminate all alternative explanations. In order to achieve this ambitious aim, the present study focuses on the Lophotrochozoan snail, *Biomphalaria glabrata*, in which innate immune memory was recently reported. We provide herein the first evidence that a shift from a cellular immune response (encapsulation) to a humoral immune response (biomphalysin) occurs during the development of innate memory. The molecular characterisation of this process in *Biomphalaria/Schistosoma* system was undertaken to reconcile mechanisms with phenomena, opening the way to a better comprehension of innate immune memory in invertebrates. This prompted us to revisit the artificial dichotomy between innate and memory immunity in invertebrate systems.

## Author Summary

Schistosomiasis is the second most widespread tropical parasitic disease after malaria. It is caused by flatworms of the genus *Schistosoma*. Its life cycle is complex and requires certain freshwater snail species as the intermediate host. Given the limited options for treating *S*. *mansoni* infections, much research has focused on a better understanding of the immunobiological interactions between the invertebrate host *Biomphalaria glabrata* and its parasite *S*. *mansoni*. Recently, we demonstrated the existence of a time-dependent and genotype-dependent acquired innate immune memory in *B*. *glabrata* snails. A primo-infection of the Lophotrochozoan vector snail, *Biomphalaria glabrata*, with *Schistosoma mansoni* totally protected the snail against a secondary challenge. Learning more about the immunobiological interactions between *B*. *glabrata* and *S*. *mansoni* could have important socioeconomic and public health impacts by changing the way we attempt to eradicate parasitic diseases and prevent or control Schistosomiasis in the field.

## Introduction

The environment of an invertebrate is filled with complex and changing populations of microorganisms, including potential pathogens. This engenders selective pressures comparable to those experienced by Gnathostomes [1], and means that invertebrates should possess sophisticated immune systems capable of dealing with such pathogens. Indeed, recent studies have shown that the immune defenses of invertebrates are more complex and specific than previously thought, and the existence of innate immune memory or priming has been suggested [2–6].

To date, the observations of invertebrate innate immune memory have been mainly phenomenological and based on ecological or phenotypic studies, and little work has addressed the potential molecular and cellular mechanisms underlying these processes. The innate immune system of invertebrates includes the barrier functions of the epithelium, the cellular immune response and the humoral defense response [7,8]: the former refers to mucosal immunity and hemocyte responses (phagocytosis, encapsulation, melanization), while the latter includes pathogen recognition receptors (PRRs), antimicrobial peptides, coagulation, and the production of cytolytic molecules or reactive intermediates of oxygen and nitrogen [7,8]. During the primary infection of an invertebrate, the pathogen is recognized at the first encounter and the cellular and humoral defense responses are coordinated to neutralize the intruder. To the best of our knowledge, the existing studies investigating the molecular mechanisms of innate immune memory in invertebrates have all suggested that the cellular immune response and/or hemocyte phagocytosis is/are improved upon a subsequent encounter with the same pathogen [9–11]. For example, in *Porcellio scaber*, enhanced phagocytosis was demonstrated after a first encounter with different bacteria species (*Bacillus thuringiensis/Escherichia coli)* [12]. For *Anopheles gambiae*, hemocytic differentiation evidenced by increased mRNA levels of hemocyte-specific genes, such as thioester-containing protein 1 (TEP1) and leucine rich repeat immune protein 1 (LRIM1) was reported following initial exposures to *Plasmodium* and bacteria [5]. In shrimp, bacterial challenge was followed by an enhanced cellular immunity characterized by a significant increase in the percentage of phagocytic cells [13]. The innate immune memory response in invertebrates has been previously described as being involved in two mechanisms, namely:

- a process of acquired resistance or sustained response that consists of a long-lasting protection against a later challenge that persists even if the pathogen is neutralized; or
- a recalled response that consists of the ability to store information of previously met pathogens and recall it later to generate a faster and more powerful response against a subsequent exposure to the same pathogen.

However, the international community working on invertebrate innate immunity believes that the existing innate immune memory observations cannot be used in isolation. Instead, they should be used to construct hypotheses that need now to be exhaustively tested with rigorous functional, cellular, biochemical and molecular methods, until all alternative explanations are eliminated [10,14]. To fully understand the capabilities of invertebrate immune systems, we must use global molecular approaches at the whole-organism level to investigate the mechanisms that form the basis for innate immune memory, while being guided by the existing observations and phenotypic descriptions. Without this demonstration, the existence of immune memory process in invertebrates will remain controversial and doubted by many immunologists [for the polemic, see [14,15]].

Recently, we demonstrated that a primo-infection of the Lophotrochozoan vector snail, *Biomphalaria glabrata*, with *Schistosoma mansoni* protected completely the snail against a homologous secondary challenge [4]. Total protection was reached 10 days after primo-infection, and was maintained for the rest of the snail’s lifespan. Our findings provided evidence for the existence of a time-dependent acquired innate immune memory in *B*. *glabrata* snails [4,16]. Moreover, we used homologous and heterologous challenges to demonstrate that this innate immune memory was genotype-dependent, in that the protection decreased with increasing neutral genetic distance between the parasites used for the primo-infection and the secondary challenge [4].

Here, we sought to investigate the molecular mechanisms of innate immune memory in *B*. *glabrata* in response to *S*. *mansoni* infections. We precisely describe the snail’s immune response phenotypes, then characterize the molecular determinants involved in *B*. *glabrata* innate immune memory via a global transcriptomic approach and a targeted proteomic analysis of plasmatic factors performed in combination with RNA interference. This work provides the first global investigation of the molecular processes supporting innate immune memory in an invertebrate model. Moreover, *Biomphalaria* snails have an important role in the transmission of Schistosomiasis the second most widespread human parasitic disease after malaria causing 200,000 deaths annually. More emphasis on snail-related research, on the role of snails and parasite intramolluscan larval stages in transmission is essential for changing the way we attempt to eradicate parasitic diseases [17,18]. Learning more about the immunobiological interactions between *B*. *glabrata* and *S*. *mansoni* could have important socioeconomic and public health impacts by contributing to the discovery of new ways to prevent and/or control Schistosomiasis diseases by limiting the parasite in the field.

## Materials and Methods

**Fig 1:**
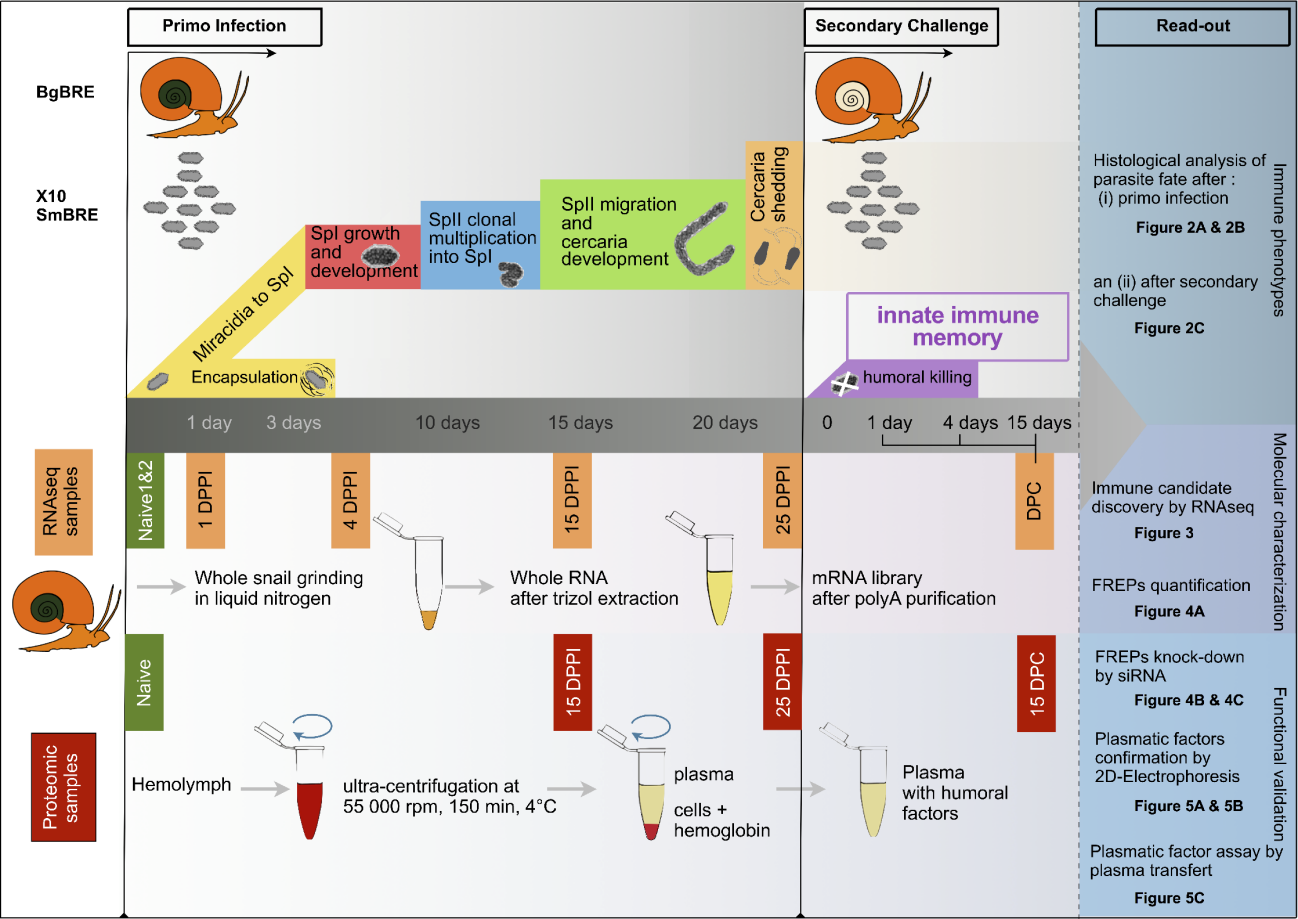
Overview of experimental procedures. Innate immune memory experiments were carry out. For primo-infection, Brazilian *Biomphalaria glabrata* (BgBRE) snails were individually exposed to 10 miracidia of their sympatric Brazilian *Schistosoma mansoni* trematode parasite (SmBRE). Following infection depending on the compatibility status of the snail/parasite couples, some of the miracidia were encapsulated by the hemocytes (snail immune cells) or developed into primary sporocysts (intra-molluscan stage of the parasite). Intramolluscan parasite stages include two generations of sporocysts (primary sporocyst (SPI) and secondary sporocyst (SPII)) and the production of cercariae. SPII developed inside SPI and migrated to reach the snail hepato-pancrea. Cercariae developed inside SPI and migrated back into the snail to reach the aquatic environment. Twenty-five days after primo-infection, the snails were challenged for a second time with again 10 SmBRE miracidia. In this case all miracidia degenerated in snail tissues, demonstrating the activation of a humoral immune response. Immune phenotypes observed during innate immune memory process were analyzed using a histological approach (see Fig 2A, 2B & 2C). In order to explore the molecular mechanisms of innate immune memory several experimental procedures were designed. A RNAseq experiment was realized with samples recovered from uninfected snails (Naive 1, Naive 2), samples recovered at 1, 4, 15 and 25 days post primo-infection (DPPI) and at 1, 4 and 15 days post-secondary challenge (DPC) (see Fig 3). Based on RNaseq results, functional validation of the FREP immune recognition receptor was undergone. First, individual quantification were made for all FREPs annotated on transcriptomic analysis (see Fig 4A). FREP knockdown was then carried out by siRNA injection, normalized by siGFP and monitored by Q-RT-PCR (see Fig 4B & 4C). Finally, to confirm the involvement of plasmatic factors in innate immune memory, snail hemolymph was recovered (Naive, 15, 25 DPPI and 15 DPC) and plasmatic fraction was characterized by 2D-gel electrophoresis (see Fig 5A & 5B). Plasma samples were also injected to naïve snails to demonstrate that immune protection could be acquired following primed snail plasma transfer (see Fig 5C).

Further details are provided in S1 Appendix and Fig 1.

### Snail and parasite strains

The present work utilized a strain of *Biomphalaria glabrata* originating from Brazil (BgBRE), along with its 100% compatible sympatric strain of *Schistosoma mansoni* (SmBRE). Both were as previously described [19].

### Innate immune memory protocol

One hundred and forty BgBRE snails were primo-infected with 10 miracidia of SmBRE each; 60 were secondarily challenged 25 days later with 10 miracidia of SmBRE each. For the RNAseq approach, pools of 20 BgBRE snails were recovered at 1, 4, 15, and 25 days post primo infection (DPPI); these were designated 1DPPI, 4DPPI, 15DPPI and 25DPPI, respectively. Twenty more snails were recovered at each of 1, 4 and 15 days after the secondary challenge; equimolar amounts of each of these experimental groups were joined together into a single sample designated as days post-secondary challenge (DPC) sample. Two pools of 20 uninfected snails (designated naive1 and naive2) were sampled and used as control replicates. For the proteomic approach, 200 BgBRE snails were primo-infected and secondarily challenged with 10 miracidia (per round) of SmBRE. Plasma sampling was performed on the same schedule described for the RNAseq experiments. Five biological replicates (5 independent pooled plasmas from 10 snails) were recovered from naïve snails (controls), and 15DPPI, 25DPPI and 15DPC snails.

### Histological procedures

Forty-eight hours after primo-infection or secondary challenge with 10 miracidia of SmBRE, snails were fixed in Halmi’s fixative (mercuric chloride 4.5%, sodium chloride 0.5%, trichloroacetic acid 2%, formol 20%, acetic acid 4% and 10% of picric acid-saturated aqueous solution). Fixed mollusks were dehydrated and embedded in paraffin as previously described [20,21]. Transverse histological sections (10-μm thick) were cut and stained using azocarmine G and Heidenhain’s azan. The following serial steps were used: (i) re-hydration (toluene, 95, 70, 30% ethanol and distilled water); (ii) coloration (azocarmine G, 70% ethanol / 1% aniline, 1% acetic alcohol, distilled water, 5% phosphotungstic acid, distilled water, Heidenhain’s azan) and (iii) dehydration (95% ethanol, absolute ethanol, toluene). The preparations were then mounted with Entellan (Sigma Life Science, St. Louis Missouri, USA) and subjected to microscopic examination.

### Whole-body RNA sequencing

Total RNA was extracted from naive1, naive2, 1DPPI, 4DPPI, 15DPPI, 25DPPI and DPC samples using nitrogen and the TRIzol reagent (Sigma Life Science, St. Louis Missouri, USA). mRNAs were sequenced in paired-end, 72-bp read length, with three samples multiplexed per lane, using an Illumina Genome Analyzer 2 (Montpellier Genomix (MGX), Montpellier, France).

### Transcriptome assembly

*De novo* transcriptomes were assembled using high-quality reads (phred > 29) from all seven sequenced samples and an in-house pipeline created using the Velvet (v1.2.01), Oases (v0.2.04) and CDhit EST (v4.5.4) programs [22]. A consensus reference transcriptome was optimized using various parameters, including k-mer length, insert length and expected coverage, as previously described [22]. To suppress non-molluscan transcripts, a BLAST-based transcriptome subtraction strategy was used. Transcripts were compared against the *B*. *glabrata* draft genome, and transcripts with identities and coverages of less than 80% were deleted. This led to a final subtraction of 45.1% of the transcripts, most of which corresponded to *S*. *mansoni*. The final size of the transcriptome was 159,711 transcripts.

### Differential expression analysis of transcripts

Quality reads (Phred score >29) were aligned to the assembled transcriptome using the C++ script, Bowtie2 (v2.0.2) (mapping quality score 255), which was run locally using the Galaxy Project server [23]. The DESeq2 software [24] (v2.12; http://www.bioconductor.org/packages/release/bioc/html/DESeq2.html) was run under the default settings to compare duplicate samples from uninfected snails (naive1 and naive2) versus infected samples to quantify differential gene expression (P value < 0.1). A heatmap was constructed to analyze transcript expression patterns (log2 fold change) using Hierarchical Ascending Clustering (HAC) with Pearson correlation, as applied by the Cluster 3.0 [25] and JavaTreeView software packages. We used quantitative real time PCR (Q-RT-PCR) to ascertain RNAseq results. For that purpose the correlation between the rpkm (reads per kilobase per million mapped reads) of RNAseq and the ct (cycle threshold) of Q-RT-PCR was tested and confirmed (see S1 Fig). Primer sequences used in Q-RT-PCR were available in S3 Table.

### FREP RNA interference

Small interfering RNAs (siRNAs) were used to knock down FREP2 (GenBank accession number gi|16303186), FREP3 (gi|18389116) and FREP4 (gi|16303188). Three siRNA duplexes (Eurogentec) were used in conjunction with Invivofectamine transfecting agent (Invitrogen, CA). Two hundred ng of pooled FREP duplex siRNA or GFP siRNA (used as control) were injected into the cardiac sinus of *B*. *glabrata* snails as previously described [26]. One, Two and four days following siRNA injection, the knock-down efficiency was confirmed by Q-RT-PCR analysis of FREP2, -3 and -4 mRNA expression. For phenotypic analysis, 90 BgBRE snails were infected with 10 miracidia of SmBRE. Twenty-one days after primo-infection, these snails were individually injected with or without siFREPs or siGFP. Four days later, each snail was subjected to a secondary challenge with 10 miracidia of SmBRE. Fifteen days post-challenge, snails were fixed and the parasite prevalence was determined by identifying schistosome primary sporocysts (SpI), in snails tissue as previously described [27,28].

### Proteomic screening of primed snail plasma

Hemolymph was collected from the head-foot region of 50 BgBRE snails (5 pools of 10 snails, 5 biological replicates) at each time point of the infection process (Naïve, 15DPPI, 25DPPI, and 15DPC). Hemocytes were pelleted by centrifugation at 2500 rpm for 15 min at 4°C, and discarded. Hemoglobin was separated from the plasma by ultracentrifugation at 55,000 rpm for 2.5 h at 4°C and the supernatant plasma samples were recovered. Protein concentration of the samples were estimated using a 2D Quant kit (GE Healthcare life sciences). The plasma (5 biological replicates for each of the 4 time points) was subjected to 2D gel electrophoresis on 12% SDS-PAGE gels using 100 μg of plasma, denaturing buffer, and 17 cm IPG Strips, pH 3–10 non-linear gradient (BioRad). Gels were stained with mass spectrometry (MS)-compatible silver staining, and comparative analysis of digitized proteome maps was performed using the PDQuest 7.4.0 image analysis software (Bio-Rad). Spots showing obvious qualitative and quantitative (at least 2-fold) differences were excised from the gel and characterized by nanoscale capillary liquid chromatography on an Ultimate 3000 coupled to a LTQ-Orbitrap tandem mass spectrometer (nanoLC–MS/MS) mapped to Swiss prot-trembl and the *Biomphalaria glabrata* Brazil transcriptome (http://ihpe.univ-perp.fr/enseignement/). A protein was considered to be correctly identified if at least two peptides were confidently matched to database sequences with an overall Mascot score greater than 50 [29].

### Plasma transfer

Plasma samples were recovered from 15DPPI or naïve snails as described above. PBS-snail solution (8.41 mN Na_2_HPO_4_, 1.65 mN NaH_2_PO_4_, 45.34 mM NaCl) was used as the control injection. Twenty μl of each sample (naïve plasma, 15DPPI plasma, or TBS Tween solution) were injected into 25 BgBRE snails per group. One group of 48 BgBRE snails was left untreated and used as a control for infection. Fifteen days after injections, the snails in the four experimental groups were infected with 10 miracidia/snail of SmBRE. This period of 15 days was used to ensure that an observed phenotype was not a direct effect of the injected molecules, but rather reflected their ability to activate the snail’s immune response pathways. Fifteen days after infections, snails were dissected and the parasite prevalence was quantified as described above.

### Statistical analysis

To test for significant differences in prevalence, Fisher’s exact test was used, with P ≤ 0.05. For the proteomic approach, statistically significant quantitative differences between spots were tested using a Mann-Whitney U test, as applied with the PDQuest 7.4.0 software (BioRad).

### Ethical statement

The laboratory and experimenters possessed an official certificate of the French Ministry of National Education, Research, and Technology, CNRS and DRAAF Languedoc Roussillon for experiments on animals, animal housing, and animal breeding (# A66040; decree # 87–848, October 19, 1987; and authorization # 007083).

## Results

### Histological inspection of *B*. *glabrata* immune response phenotypes

**Fig 2:**
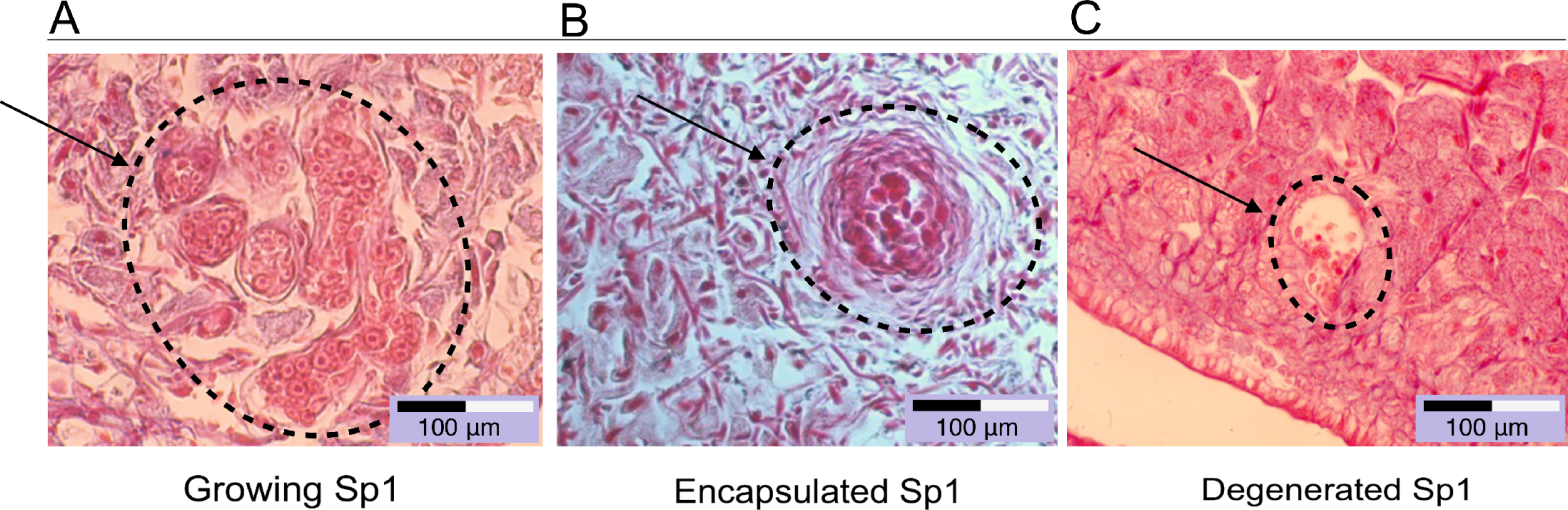
The immune response of *B*. *glabrata* to *S*. *mansoni* infection. The Brazilian strain of albino *B*. *glabrata* (BgBRE) is 100% susceptible (for 10 miracidia and upwards) to its corresponding strain of *S*. *mansoni* (SmBRE). When a snail is infected with 10 miracidia of *S*. *mansoni* within the same individual compatible and incompatible interactions occur, 3 to 4 miracidia develop normally in the snail’s tissues while the others are recognized and encapsulated by the snail’s cellular immune response. A. Six-day-old sporocyst in a compatible interaction. B. Encapsulated sporocyst 48 h after primary infection in an incompatible interaction. C. Six-day-old sporocyst in a primed snail. Primed BgBRE are 100% protected against a secondary challenge with SmBRE. Sporocysts from secondary challenge were neutralized by immune humoral factors.

Three types of immuno-biological interactions were observed following infection of *B*. *glabrata* by *S*. *mansoni*. After primo-infection, a compatible interaction was characterized by the ability of miracidia to penetrate, transform into sporocysts and develop normally in snail tissues (30 to 40% of the entering miracidia produce SPI) (Fig 2A). In an incompatible interaction, miracidia were immediately recognized, encapsulated and killed by hemocytes; in this case, a multicellular capsule could be observed surrounding the parasite (60 to 70% of the entering miracidia were killed) (Fig 2B). Finally, in primed snails subjected to a secondary challenge, the sporocysts degenerated in the snail tissues. Encapsulation was never observed nor was the accumulation of hemocytes observed near the parasite (100% of the entering miracidia were killed by humoral factors) (Fig 2C). These findings indicate that a cellular immune response was the main outcome following a primo-infection, whereas an exclusively humoral immune response was found in primed snails subjected to a secondary challenge (Fig 2).

### A whole-snail transcriptomic approach for investigating the molecular basis of innate immune memory

The RNAseq values were validated by a correlation analysis performed with our quantitative Q-RT-PCR data (S1 Fig, S3 Table). We obtained a good correlation (R^2^ = 0.768), indicating that the representations of transcripts in our RNAseq results appeared to be proportional to those obtained through Q-RT-PCR amplifications.

**Fig 3:**
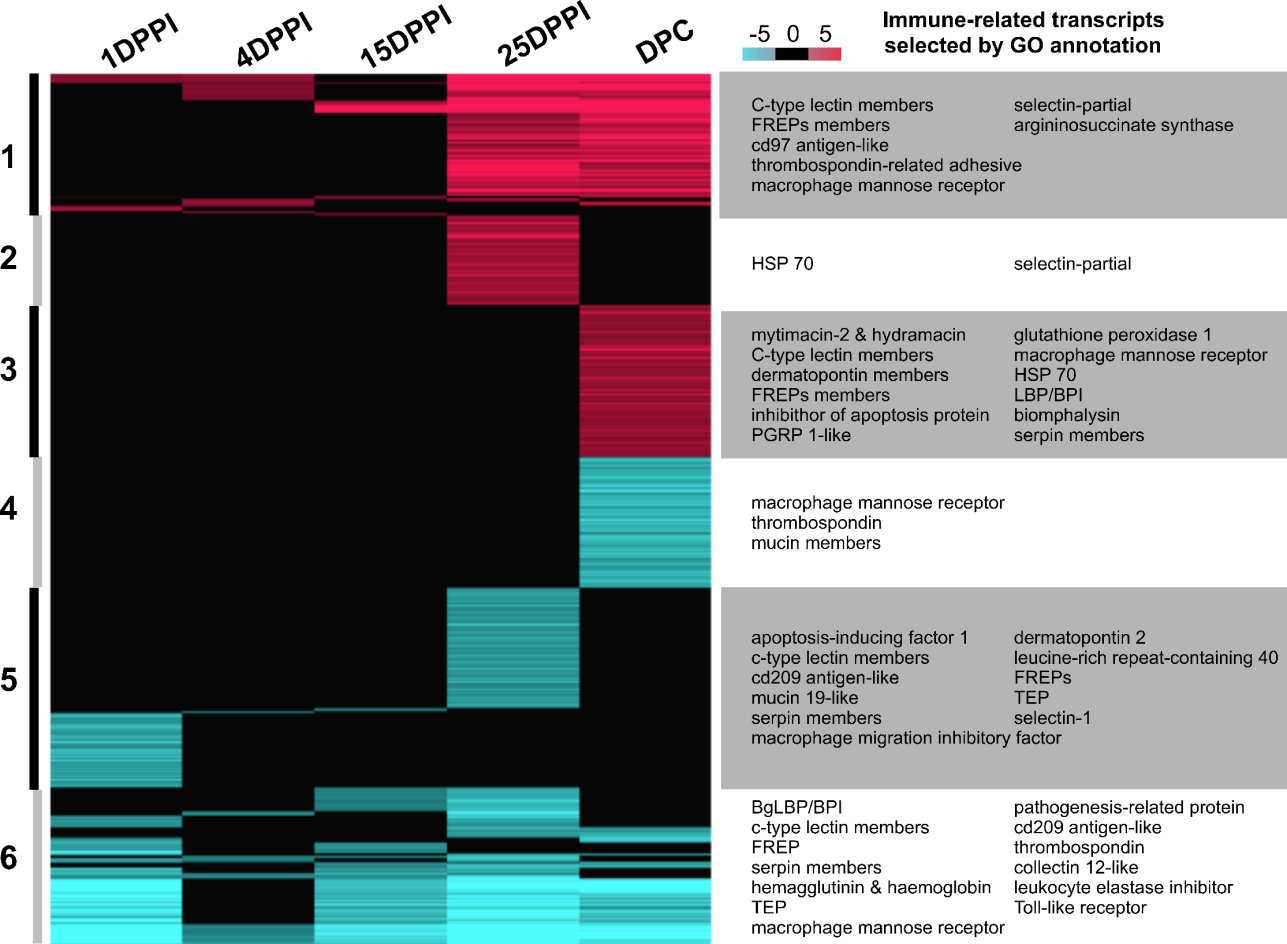
RNAseq analysis of the innate immune memory response of *B*. *glabrata* to *S*. *mansoni*. Heatmap showing differentially represented transcripts compared to naïve snails, as identified by DESeq2 analysis (p < 0.1). Color scale indicates the Log2FC ratio from under-represented (blue) to over-represented (red) transcripts. Transcripts were grouped into six clusters based on their expression patterns during the process of innate immune memory. Samples were recovered at 1DPPI, 4DPPI, 15DPPI and 25DPPI following primo-infection. Following secondary challenge samples were recovered at 1 day, 4 days and 15 days and pooled into DPC sample. Six clusters are identified: Cluster 1: transcripts over represented more than once all along infection and challenge. Cluster 2: transcripts exclusively over represented in single one condition. Cluster 3: transcripts exclusively over represented after immune challenge (DPC). Cluster 4: transcripts exclusively under represented after immune challenge (DPC). Cluster 5: transcripts exclusively under-represented in single one condition. Cluster 6: transcripts under represented more than once all along infection and challenge. FREP: Fibrinogen-related protein, HSP: Heat-shock protein, PGRP 1-like: Pathogenesis-related protein 1-like, LBP/BPI: lipopolysaccharide-binding protein/bactericidal/permeability-increasing protein, BgLBP/BPI: *Biomphalaria glabrata* LBP/BPI, TEP: thioester-containing protein.

This RNAseq experiment allowed us to identify 1887 differentially expressed transcripts, most of which were found in the 1DPPI, 25DPPI and DPC samples (Fig 3, S1 Table). At 1DPPI, most of the differentially expressed transcripts were under-represented (Fig 3). This could reflect the immuno-suppression/modulation induced by *S*. *mansoni* parasites within the first hours of an infection, when the parasites develop their molecular mimicry strategy [30,31]. At 25DPPI and DPC, the proportions of over- and under-represented transcripts were more comparable (Fig 3). Hierarchical clustering was used to sort the differentially represented transcripts into six clusters, three each corresponding to over-represented transcripts (clusters 1–3) and under-represented transcripts (clusters 4–6) (Fig 3, S1 Table). Cluster 2 included transcripts that were specific to the primo-infection and not activated following the secondary challenge. Clusters 1 and 3 appeared to be the most promising possible sources of innate immune memory candidates. Cluster 1 comprised transcripts that were over-represented following primo-infection and remained highly expressed throughout infection (sustained response); it included immune molecules known to be involved in the cellular immune response (macrophage mannose receptors, thrombospondin), as well as certain pathogen recognition receptors (PRRs) (selectins and C-type lectins). In contrast, cluster 3 comprised transcripts that were over-represented exclusively following the secondary challenge (Fig 3), including certain PRRs (FREPs, C-type lectins) and circulating immune effectors (anti-microbial peptides such as mytimacin and LBP/BPI, and biomphalysin). Interestingly, clusters 4, 5, and 6, which contained the under-represented transcripts, included immune molecules belonging to the same immune functional groups represented in clusters 1 to 3 (Fig 3). This may indicate that there is a trade-off among the immune variants involved in the specific response to *S*. *mansoni* parasites, with some members of the same family (i.e., variants or isoforms) being down-regulated to permit the over-expression of other variants. In cluster 4, notably, most of the under-represented transcripts were involved in the cellular and epithelial immune response (e.g., macrophage mannose receptors, extracellular matrix compounds, mucins). This indicates a down regulation of epithelial (mucosal immunity) and cellular immunity two major components of innate immune response following secondary challenge. The complete list of differentially represented transcripts is presented in S1 Table.

### Loss-of-function, FREP-mediated RNA interference

**Fig 4:**
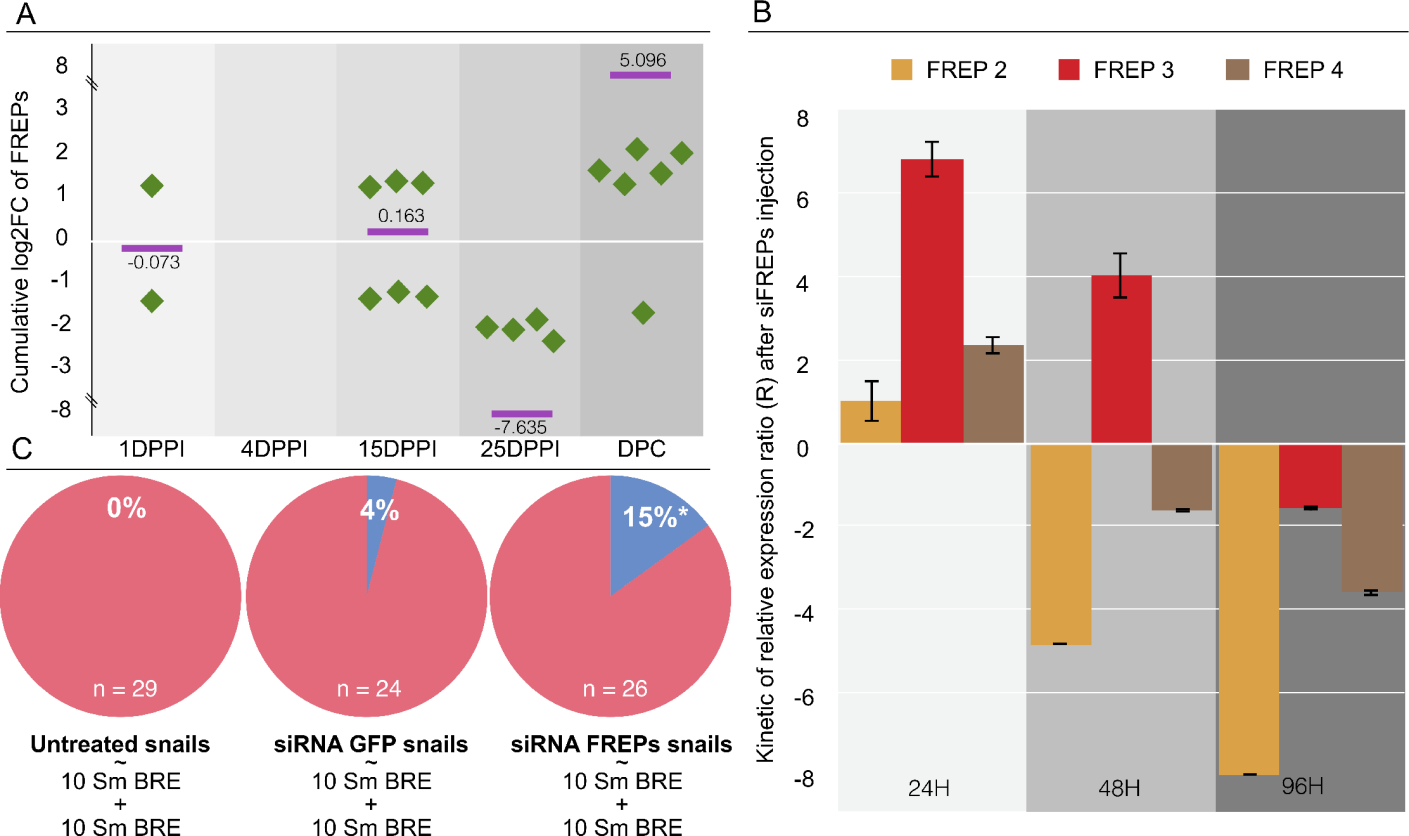
FREPs knock-down mediated by RNA interference. A. Cumulative expression [Log2FC (fold change) from the DESeq2 analysis] of FREP transcripts showed that FREPs were over-represented after the secondary challenge (DPC; 5.096 log2 fold change enrichment of FREPs transcripts). Green points corresponded to the differentially represented FREPs transcripts in each samples. Purple bars represent the cumulated Log2FC of FREP transcripts. At 4DPPI no FREP transcripts were differentially expressed, thus no value appeared in the graph. B. siRNA injection against FREP2, FREP3 & FREP4 was carried out and mRNA abundance was monitored during 4 days by Q-RT-PCR. Snails were injected with siRNAs against FREP 2, 3, and 4 or GFP (control), the relevant mRNA levels were assessed following normalization with respect to the S19 gene in siGFP injected snails versus siFREPs injected snails. Knock-down of the three FREPs tested was confirmed at 96h. C. Naïve *B*. *glabrata* and siFREP-injected snails were subjected to a typical priming experiment: Snails were infected with 10 miracidia of *S*. *mansoni* as a primo-infection, 21 days later they were injected with siGFP, or SiFREP or not treated and 4 days later they were infected with another 10 miracidia as a secondary challenge. FREP siRNA-injected snails show a significant proportion of non-primed snails (15%; *, binomial test, P < 0.05).

To estimate total FREP expression (Fig 4A) over the entire innate immune memory process based on our RNAseq data, we summed the fold changes for all the differentially represented FREP transcripts (identified in Fig 3). A huge induction (5.096-fold) of FREPs was observed following the secondary challenge (DPC, Fig 4A). SiRNA-mediated knockdown of FREPs were used in combination to ascertain the role played by these FREP candidates in innate immune memory. Knockdown of FREPs was confirmed by Q-RT-PCR; the results were normalized with respect to the mRNA expression levels in naïve snails, and compared to the corresponding levels in siGFP-control-injected snails (Fig 4B). The best knockdown level was reached at 96 h post-siRNA injection, when we observed a decrease of 7.98-, 1.56- and 3.60-fold change for FREP 2, 3 and 4, respectively (Fig 4B). The effect of FREP knockdown on the innate immune memory phenotype was assessed in a typical homologous innate immune memory assay. In untreated snails, the parasite prevalence after secondary challenge was 0% (Fig 4C), indicating that innate immune memory had effectively protected them against a subsequent exposure to *S*. *mansoni*. Injection of siGFP did not significantly change the parasite prevalence after secondary challenge (4%; Fig 4C). Following injection of the FREP siRNA, however, 15% of the snails became infected with *S*. *mansoni* following secondary challenge (Fisher’s exact test P < 0.05) (Fig 4C). Thus, FREP knockdown partially altered the innate immune memory phenotype, rendering primed snails more susceptible to *S*. *mansoni* infection.

### Global proteomic comparison of primed snail plasma

**Fig 5:**
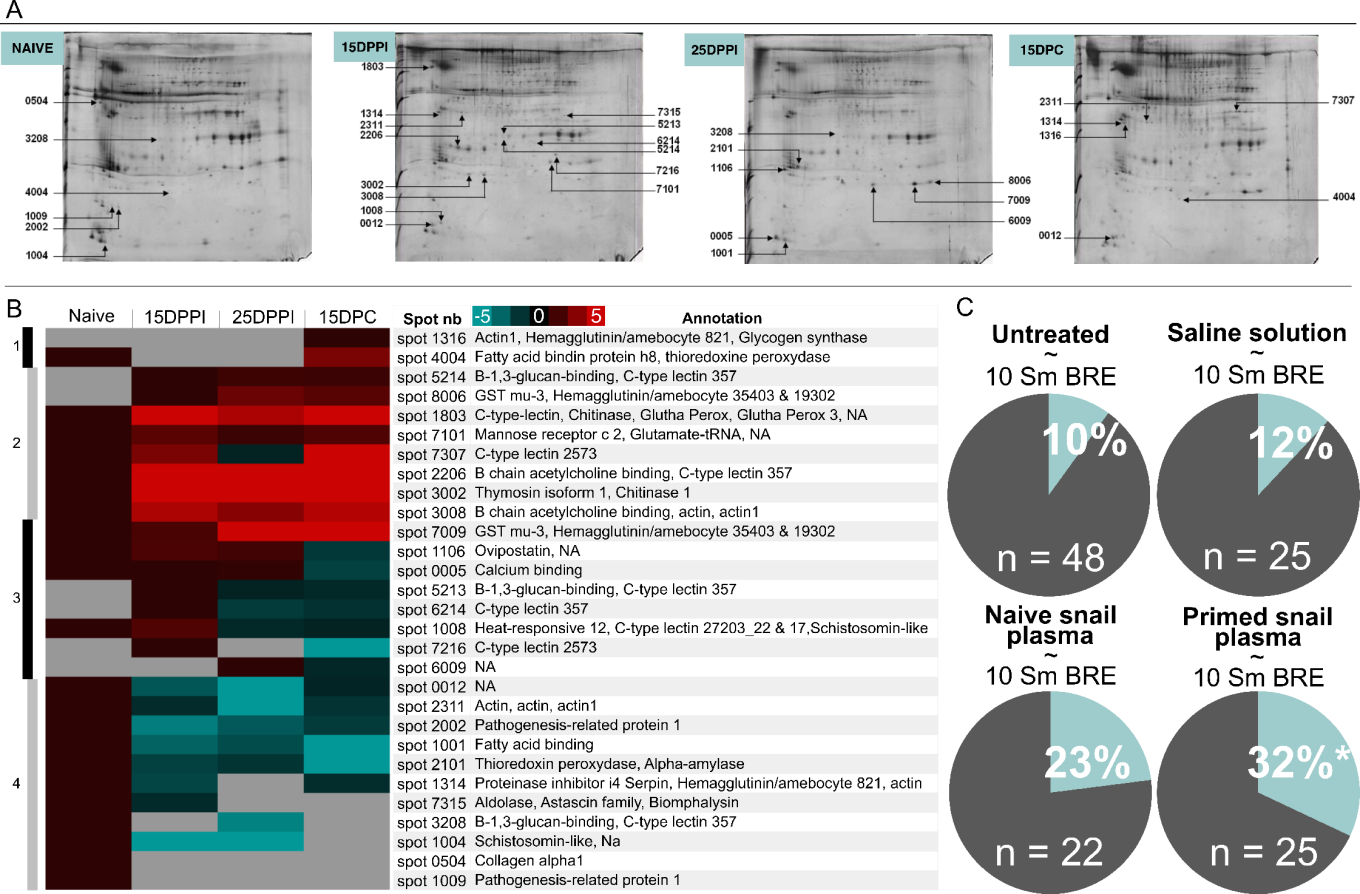
Role of *B*. *glabrata* plasmatic factors in innate immune memory response. A. 2D gel electrophoresis of plasma proteins. One gel of each plasma sample analysed was shown. Spot numbers of qualitative and quantitative differences were indicated. Four plasma samples were analysed from naïve (uninfected snails), 15DPPI and 25DPPI (recovered at 15 and 25 days after primo-infection) and 15DPC (recovered at 15 days after secondary challenge). B. Heat-Map of the qualitative and quantitative ratio versus naïve sample. Ratios were calculated using PDQuest software between all differentially regulated spots. Blue to red scale indicate ratio values from lower to higher represented spots. Four clusters are identified: Cluster 1: higher-represented proteins exclusively following secondary challenge (15 DPC). Cluster 2: sustained response: higher-represented proteins after the primo-infection and secondary infection. Cluster 3: higher-represented proteins at 15DPPI and thereafter down regulated at 25DPPI and 15DPC. Cluster 4: lower-represented proteins. C. Plasma transfer and effect on prevalence of *S*. *mansoni* infection. Four conditions were tested: untreated snails (Control group, n = 48); saline injected snails (control of injection, n = 25); naïve-plasma injected snails (n = 22); and primed-plasma injected snails (n = 25). For all the experimental groups, 15 days following injection, snails were infected with 10 miracidia of SmBRE. * indicated significant differences (P< 0.05).

To validate our RNAseq results, we focused our attention on the plasmatic compartment of *B*. *glabrata*, which should contain the actors of the humoral innate immune memory response. We performed global comparative 2D gel electrophoresis on plasma samples obtained from naive, 15DPPI, 25DPPI, and 15DPC snails. Our quantitative and qualitative bioinformatic analyses identified 29 differentially expressed spots corresponding to 62 different proteins (Fig 5A, S2 Table).

A hit map of the quantitated expression ratios was generated for the entire infection process (Fig 5B, S2 Table). Four clusters were identified (Fig 5B): the proteins in cluster 1 were up-regulated following the secondary challenge; those in cluster 2 were up-regulated after the primo-infection and remained highly expressed throughout infection (sustained response); those in cluster 3 were up-regulated after the primo-infection and down-regulated following secondary challenge those of cluster 4 were down-regulated. The differentially expressed proteins were also subjected to functional classification based on their putative functions and/or structural features. Five functional groups were identified: innate immune response proteins (receptors, effectors, and regulators), scavengers of reactive oxygen species, cell signaling proteins, gametogenesis-related proteins, and glycolysis-related proteins. Thirty seven percent of the identified molecules belonged to the innate immune response group (23 of 62 proteins) (Fig 5B). Consistent with our RNAseq results, we observed an important enrichment of potential humoral immune candidates in the plasma of primed snails. The molecules involved in immune recognition and opsonization included numerous isoforms belonging to different families of circulating immune receptors that were also identified in our transcriptomic analysis (e.g., C-type lectins, β-1,3-glucan binding protein, and hemagglutinin/amebocyte aggregation factors). Most of these isoforms were found in clusters 1 and 2, suggesting that they may actively contribute to the innate immune memory response (Fig 5B). Among the immune effector molecules, chitinase (spot 3002, Fig 5B), fatty acid binding protein (spots 4004 and 1001, Fig 5B), and biomphalysin (spot 7315, Fig 5B) were found in clusters 1, 2 and 4, respectively. Biomphalysin was over-represented following primo-infection and secondary challenge in the transcriptomic data, but down regulated at 15DPPI and 15DPC in our proteomic data (cluster 4, spot 7315; Fig 5B). Thus, it seems that biomphalysin is highly transcribed during the innate immune memory response against *S*. *mansoni* parasites, but is thereafter consumed at the protein level. Some known regulators of the immune response (e.g., thymosin, spot 3002; serpin, spot 1314; and β chain acetylcholine binding protein, spots 2206 and 3008) were associated with protein clusters 2 and 4 (Fig 5B). In addition, other proteins known to be involved in host/parasite interactions were identified as being differentially expressed, including some anti-oxidant molecules, which were identified in protein clusters 1 and 2 (e.g., glutathione peroxidase 3 precursor, spot 1803; glutathione-S-transferase mu 3-like, spots 7009 and 8006; and thioredoxin peroxidase, spot 4004; Fig 5B).

Proteins involved in gametogenesis were also identified by our global proteomic analysis. They included schistosomin (spot 1004), which is involved in the regulation of gametogenesis [32]; pathogen-related protein (spots 2002 and 1009), which is a seminal fluid protein [33,34]; and ovipostatin (spot 1106), which is involved in oviposition and hatching [35]. These proteins were found in clusters 3 and 4, and were thus down-regulated following primo-infection and/or secondary challenge (Fig 5B) (see S2 Appendix for details).

### Plasma transfer

A plasma experiment was carried out to confirm the role played by humoral factors in innate immune memory. Plasmas were recovered from naïve or 15DPPI snails and injected into naïve snails. The snails were subjected to infection 15 days post-plasma-injection, and analyzed for phenotypes at 15 days post-infection (Fig 5C). Injection of saline solution has no significant effect on the prevalence of parasitic infection compared to the untreated control (88% and 90%, respectively) (Fig 5C). Transfer of naive snail plasma also failed to have any significant effect (prevalence of 77%) (Fig 5C). However, a statistically significant decrease in the prevalence of infection was observed for snails injected with primed snail plasma (from 90% to 68%; P = 0.04). This protection remained efficient at 15 days post-transfer, confirming that plasmatic humoral factors function to activate the immune system and prepare the snail to answer a subsequent encounter with the parasite.

## Discussion

Innate immune memory, which is a process through which an organism acquires a (more or less) specific and long-lasting protection against later challenges that persists even after the pathogen is neutralized, has been described in diverse invertebrate phyla [2,3,5,6,14,36–39]. This suggests that innate immune memory exists in a broad range of invertebrates.

A form of innate immune memory was previously demonstrated in *B*. *glabrata* in response to *S*. *mansoni* infection, and shown to be dependent on the genotype of the pathogen [4]. Here, we provide evidence that innate immune memory in *B*. *glabrata* is not associated with an enhancement of the cellular immune response, as has been suggested in other invertebrate species [5,12,13]. Instead, our histological analysis (Fig 2) revealed that in primed snails, the parasites of a secondary challenge fail to develop into sporocysts and are killed by the host, without any observable cellular immune response/hemocytic reaction. Together, these findings show that, after a primo-infection, each successive encounter with a similar parasite initiates an exclusive humoral immune defense response in *B*. *glabrata*.

Accordingly, we set out to identify the humoral factors through which primed snails are able to neutralize the sporocysts of a secondary challenge. A whole-snail RNA sequencing approach was conducted to identify molecular candidates that might be involved in the innate immune memory response. For RNAseq approach the cluster 1 (sustained-response transcripts) and cluster 3 (secondary challenge-specific transcripts) contained the most promising candidates (Fig 3). Numerous circulating or cell-surface PRRs were found. These immune receptors, which recognize terminal sugar residues on the glycans that are attached to the surface proteins of some microorganisms, act in the pathogen recognition and clearance processes of innate immunity. The identified PRRs belong to various families, including the macrophage mannose receptors, selectins, C-type lectins and fibrinogen-related proteins (FREPs). Cytotoxic and cytolytic molecules were also identified, as were anti-oxidant molecules. The latter finding suggests that the reactive oxygen species pathway may be activated as part of the humoral innate immune memory response of *B*. *glabrata*. Moreover, RNAseq cluster 4 (molecules down-regulated upon secondary challenge) included transcripts whose products are involved in the epithelial and cellular immune response, such as macrophage mannose receptors, extracellular matrix compounds and mucins. This strengthens our contention that there is a shift from cellular or mucosal immunity to humoral immune response during the innate immune memory response in *B*. *glabrata* snails.

An unexpected result of this study was the identification of RNAseq cluster 3 and proteomic cluster 1. Until now, innate immune memory was believed to be the result of two potential regulatory processes: (i) a sustained immune response consisting of the long-lasting up-regulation of immune molecules after a primo-infection (as observed in RNAseq cluster 1 and proteomic cluster 2); or (ii) a recalled response that consists of the ability to store information and recall it later for a faster and more powerful response against subsequent pathogenic exposure (never been observed in any of the RNAseq or proteomic clusters) [36,39]. Here, we provide the first report of a novel unexpected regulatory process for invertebrate innate immune memory, in which transcripts are specifically up regulated following the secondary challenge (RNAseq cluster 3, proteomic cluster 1) without having been previously activated by the primo-infection. This new observation warrants further study, such as through the identification of its potential molecular hallmarks, including the genetic (activation of transcription factors) and/or epigenetic (DNA methylation, chromatin markers, lncRNA, miRNA, etc.) factors that form the basis for this exclusive secondary challenge innate immune memory response.

To validate the potential roles played by the identified molecular candidates in the observed innate immune memory phenotype, we performed three sets of experiments: we used RNAi to knockdown certain FREPs and examined the priming response; we characterized the snail plasma proteome; and we examined the effect of injecting naïve snails with plasma from primed snails.

As FREP lectins showed extensive differential representation following the secondary challenge (see Fig 4A), and they are known to play key roles in the immunobiological interactions between *B*. *glabrata* and *S*. *mansoni*, they were chosen as candidates for RNAi invalidation. The FREPs comprise one or two amino-terminal immunoglobulin domains (IgSF) and a carboxyl-terminal fibrinogen domain (FBG). They belong to a multigene family of at least 14 members, and undergo somatic rearrangements leading to remarkable diversification [40,41]. FREPs can precipitate soluble antigens derived from trematodes. Their expression levels increase in response to infection with *S*. *mansoni* [42,43], and they form immune complexes with similarly highly polymorphic and individually variable mucins (the *Sm*PoMucs) that act as antigens of *S*. *mansoni* [44]. The high level of diversification within FREP family members results in a huge proportion of partial FREP transcript sequences generated by the RNAseq *de novo* assembly [22]. We were unable to precisely identify the FREP variants/isoforms present in the various RNAseq clusters. Thus, to confirm the role of FREPs in innate immune memory, we invalidated FREP 2, 3, and 4, as they had previously been demonstrated to be involved in the response of *B*. *glabrata* to trematodes. More specifically, FREP 2 is involved in immune complexes [44]; FREP 3 knock-down reverts the snail resistance status to trematodes [45]; and microarray analysis showed that FREP 4 is over-expressed following infection by *S*. *mansoni* [43]. SiRNA-mediated knock-down of FREP 2, 3 and 4 (Fig 4B) was found to reduce the innate immune memory phenotype by 15% (Fig 4C). These results indicate that these FREPs are involved in *B*. *glabrata* innate immune memory, but demonstrate also that additional molecular partners (and/or other FREP variants) also play roles in the pathogen recognition and innate immune memory of this snail.

Our characterization of the plasmatic proteome confirmed that most of the molecules identified in the RNAseq data were present and differentially expressed at the protein level. The presence of these molecules in snail plasma also confirms that they act as circulating humoral factors. The most promising candidates for participation in innate immune memory were found in protein cluster 1 (secondary challenge-specific proteins) and protein cluster 2 (sustained-response proteins) (Fig 5). Numerous PRRs were identified (e.g., macrophage mannose receptors, C-type lectin, hemagglutinin, and β -1, 3-glucan binding proteins). Interestingly, we did not recover any FREP in this proteomic analysis of primed snail plasma. We speculate that the FREPs were lost from our analysis due to their precipitation with parasitic antigens [44]. Alternatively, and as suggested previously for other families of highly variable molecules [46], the different variants may be expressed at such low levels that they are not visible on 2D gel electrophoresis. Indeed, we recently demonstrated that FREPs are expressed at a low level compared to other PRRs in naive *B*. *glabrata* snails [22]. Plasmatic proteome was also composed of cytotoxic and cytolytic molecules, including antimicrobial peptides [AMPs; e.g., hydramicin, mytimacin and lipopolysaccharide binding protein/bactericidal permeability-increasing protein (LBP/BPI)], and the biomphalysin. We recently reported the molecular cloning and functional characterization of the *B*. *glabrata* biomphalysin and its involvement in the killing of *S*. *mansoni* [47]. Biomphalysin, which shares a common architecture with proteins belonging to the aerolysin superfamily, is strictly expressed in immune-competent cells. Recombinant biomphalysin was shown to bind to parasitic membranes and exhibit cytotoxic activity toward *S*. *mansoni* sporocysts. Our RNAseq and proteomic analyses primarily recovered immune recognition factors, many of which had multiple isoforms. This suggests the involvement of both translational and post-translational regulation. Additional work is now needed to clarify the functions of these molecules in the humoral innate immune memory response of *B*. *glabrata*. For example, C-type lectins are known to be involved in the innate immune memory responses of mollusks; their expression levels increased in the scallop, *Chlamys farreri*, following vaccination with heat-killed *Vibrio anguillarum*, and successive challenges with *V*. *anguillarum* or *Micrococcus luteus* enhanced this protection [11]. In addition to their roles in pathogen recognition and opsonization, lectins also possess direct cytotoxic activities. Examples of cytotoxic lectins include plant-derived ricin from *Ricinus communis* beans [48], the fungus-derived N-acetyl-D-galactosamine-specific lectin from *Schizophyllum commune* [49], and the invertebrate-derived hemolytic lectin, CELIII, from *Cucumaria echinata* [50]. Thus, it seems reasonable to hypothesize that some of the lectins identified in the present study may participate in the humoral innate immune memory response both as recognition receptors and as cytotoxic/cytolytic molecules involved in killing the *S*. *mansoni* SpIs of the secondary challenge.

Lastly, we explored whether the transfer of plasma from primo-infected snails could provide recipient naïve snails with enhanced immunity. In a previous study, plasma transfer from *S*. *mansoni*-resistant *Biomphalaria tenagophila* to susceptible snails was associated with a transfer of resistance [51]. Here, we found that transfer of primed snail plasma to naive snails significantly reduced the prevalence of *S*. *mansoni* infection by more than 20% compared to controls (Fig 5). A comparable experiment was previously performed using the mosquito, *Anopheles gambiae*, and its bacterial pathogens [5]. The transfer of cell-free hemolymph from infected mosquitoes into healthy mosquitoes triggered increases in the hemocyte populations of transferred mosquitoes, indicating that humoral factors could promote a cellular immune response that protected mosquitoes against subsequent bacterial challenges [5]. In *Biomphalaria*, plasma transfer experiments demonstrated that soluble humoral factors are released into the hemolymph of primed snails, and that the transfer of such factors activates/regulates the humoral immune response in recipients and confers enhanced antischistosomal immunity against subsequent encounters with the pathogen.

To conclude, we evidence for the first time the molecular basis of innate immune memory in an invertebrate model, and we demonstrate the role of humoral factors in such phenomenon. The previous studies addressing the molecular mechanisms of innate immune memory in invertebrates have mainly suggested the involvement of elevated hemocyte phagocytosis [9,10]. In *B*. *glabrata*, innate immune memory seems to be supported by humoral factors that trigger the degeneration/death of the parasite. Moreover the innate immune memory protection was previously shown to decrease with increasing neutral genetic distance between the parasite used for the primo-infection and the one used for the secondary challenge [4]. We hypothesized that better protection against a homologous (vs. heterologous) secondary infection involves the mobilization of specific repertoires of *B*. *glabrata* immune receptors to target certain subsets of *S*. *mansoni* genotypes. Our present findings prompt us to speculate that the genotype-dependent innate immune memory of *B*. *glabrata* may be supported by a diverse repertoire of FREPs and other PRRs (Figs 4 and 5). As previously suggested [22], PRRs might serve as collaborative recognition factors that can be processed as homologous or heterologous multimers, which then act as immune recognition complexes to increase the host’s PRR repertoire and mediating anti-pathogen responses. Once recognized, the pathogen is neutralized by the release of cytotoxic/cytolytic circulating factors. Here, we demonstrate that biomphalysin is highly transcribed following secondary challenge and consumed at the protein level during innate immune memory response, suggesting that biomphalysin may play a major role in neutralizing the pathogen following immune recognition. Clearly, future studies are needed to fully understand how these molecules mediate and regulate the specificity of the innate immune memory defense seen in *B*. *glabrata*.

To reconcile mechanisms with phenomena a molecular characterisation of innate immune memory in *Biomphalaria/Schistosoma* model was undertook. Transcriptomic, proteomic, and functional validation lead us to a new molecular comprehension of innate immune memory processes and prompted us to revisit the artificial dichotomy between innate and memory immunity in invertebrate systems.

## Supporting Information

S1 Fig Correlation between RNAseq and Q-RT-PCR. The correlations between the RNAseq reads per kilobase per million (log2 RPKM) values and the corresponding Q-RT-PCR cycle threshold (Ct) values were calculated and shown for 36 randomly selected transcripts. RPKM values were calculated using a local alignment with Bowtie2 on *de novo* assembled *B*. *glabrata* transcriptome. Cts were calculated by Q-RT-PCR using the Naive1/Naive2 biological samples previously used for RNAseq. Log2 RPKM values were an average of two biological replicates of two independent snail pools and represented in log2 scale. Horizontal lines indicate the standard error between these two points. Non-normalized Ct values were an average of three technical replicates of Q-RT-PCR. The vertical lines indicate the standard error between these three points. A dependency between variables, increasing of RPKM and decreasing of Ct is shown by least-square regression analysis and its correlation coefficient R² = 0,76804.
(TIF)

S1 Table List of differentially represented transcripts in RNAseq clusters. Quality reads (Phred score >29) were aligned on the transcriptome assembly using the C++ script Bowtie2 (v2.0.2) (255 score) running thanks local engine using Galaxy Project server (Giardine, Riemer et al. 2005). The DESeq2 software (Love, Huber et al. 2014) (v2.12;http://www.bioconductor.org/packages/release/bioc/html/DESeq2.html) (defaults settings) allows for quantifying the differential gene expression with comparing two biological duplicates from uninfected snails sample (Bre1 and Bre2) against infected samples (Pvalue<0.1). For each cluster transcript ID, Blast2GO annotation and Log2FC results were indicated.
(XLSX)

S2 Table All cluster identified in Fig 5. Ratio of expression and mapping annotation were indicated for all the sequences identified as differentially regulated in the plasma comparative proteomic approach.
(XLSX)

S3 Table Primers used for Q-RT-PCR. Identification numbers (iD), Blast2GO annotation and sequences of forward and reverse primers were indicated for 36 randomly selected transcripts of Naive1/Naive2 RNAseq biological samples. These sequences were used to analyse the correlation between the RNAseq reads per kilobase per million (log2 RPKM) values and the corresponding Q-RT-PCR cycle threshold (Ct) see S1 Fig.
(XLSX)

S1 Appendix Expanded Materials and Methods section. (DOCX)

S2 Appendix Trade-off between reproduction and immunity. (DOCX)
