# Supplementary figures and images for: A Shift from Cellular to Humoral Responses Contributes to Innate Immune Memory in the Vector Snail Biomphalaria glabrata
Source: PLoS Pathog. 2016 Jan 6;12(1):e1005361. doi: 10.1371/journal.ppat.1005361 (PMC4703209; doi:10.1371/journal.ppat.1005361)

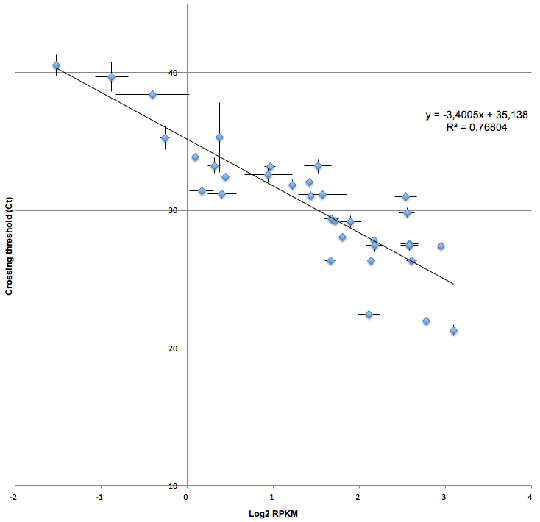

Supplement: S1 Fig — The correlations between the RNAseq reads per kilobase per million (log2 RPKM) values and the corresponding Q-RT-PCR cycle threshold (Ct) values were calculated and shown for 36 randomly selected transcripts. RPKM values were calculated using a local alignment with Bowtie2 on de novo assembled B. glabrata transcriptome. Cts were calculated by Q-RT-PCR using the Naive1/Naive2 biological samples previously used for RNAseq. Log2 RPKM values were an average of two biological replicates of two independent snail pools and represented in log2 scale. Horizontal lines indicate the standard error between these two points. Non-normalized Ct values were an average of three technical replicates of Q-RT-PCR. The vertical lines indicate the standard error between these three points. A dependency between variables, increasing of RPKM and decreasing of Ct is shown by least-square regression analysis and its correlation coefficient R² = 0,76804. (TIF) [file ppat.1005361.s001.tif]
