# Supplementary material for: A Shift from Cellular to Humoral Responses Contributes to Innate Immune Memory in the Vector Snail Biomphalaria glabrata
Source: PLoS Pathog. 2016 Jan 6;12(1):e1005361. doi: 10.1371/journal.ppat.1005361 (PMC4703209; doi:10.1371/journal.ppat.1005361)
Supplement: S1 Appendix — (DOCX) [file ppat.1005361.s005.docx]

**Appendix S1: Expanded Materials and Methods section.**

**Ethic statements**

Our laboratory holds permit # A66040 for experiments on animals from both the French Ministry of Agriculture and Fisheries, and the French Ministry of National Education, Research, and Technology. The housing, breeding and animal care of the utilized animals followed the ethical requirements of our country. The researchers also possesses an official certificate for animal experimentation from both French ministries (Decree # 87–848, October 19, 1987). Animal experimentation followed the guidelines of the French CNRS. The different protocols used in this study had been approved by the French veterinary agency from the DRAAF Languedoc-Roussillon (Direction Régionale de l'Alimentation, de l'Agriculture et de la Forêt), Montpellier, France (authorization # 007083).

## **Snail and parasite strains**

A strain of *Biomphalaria glabrata* originated from Brazil and its homopatric strain of *Schistosoma mansoni* was used in this study [19]. The Brazilian strain of albinos *B. glabrata* (BgBRE) is 100% susceptible (for ten miracidia and upwards) to its corresponding strain of *S. mansoni* (SmBRE). Inside the same snail, following infection with 10 miracidia of *S. mansoni* half of the parasites developed normally in snail’s tissues while other neighbouring larvae are recognized and killed by the snail cellular immune response.

*S. mansoni* was maintained (i) in their homopatric strain of *B. glabrata* and (ii) in hamsters (*Mesocricetus auratus*) as described previously [19]. Briefly, miracidia were hatched from eggs axenically recovered from 50-day infected hamster livers according to previously described procedures [31,52]. Livers were collected and homogenized and the eggs were filtered and washed to obtain miracidia.

Experimental protocol of homologous innate immune memory

Primary infections were performed on 200 juveniles BgBRE (5-6 mm in diameter). The snails were individually exposed for 12h to 10 miracidia of SmBRE in 5 mL of pond water. After infection 20 individuals of BgBRE were recovered at 24 hours, 96 hours, 15 days and 25 days post primary infection (these samples correspond to Brazil snails infected by Brazil parasites and were named: 1DPPI, 4DPPI, 15DPPI and 25DPPI respectively). Then, 25 days after the first infection, individual snails were challenged using 10 miracidia of SmBRE per snail. Three new pools of 20 BgBRE individuals were recovered at 24 hours, 96 hours, 15 days after this secondary challenge (these samples correspond to Brazil snails primo-infected by Brazil parasites and challenged by the same Brazil parasites, these samples were named : 1DPC, 4DPC and 15DPC respectively). These 7 samples of 20-pooled individuals were kept in liquid nitrogen until used for total RNA extractions.

As controls, two pools of 30 uninfected BgBRE snails (named Naive1 and Naive2) were recovered to serve as reference of constitutive gene expression for the RNAseq analysis.

Finally, innate immune memory phenotype was evaluated. At the end of the experiment 40 snails were dissected to evaluate the percentage of re-infection after the challenge. The efficiency of the challenge was also evaluated by infecting, 20 unprimed snails with 10 miracidia of SmBRE at the same time primary infected snails underwent challenge infection, (i.e. 25 days after primary infection). These snails were dissected to evaluate parasite infectivity and confirm the innate immune memory phenotype by quantifying the prevalence of infection as described previously [4].

**Histological procedures**

A histological approach was conducted to investigate and describe the fate of *S. mansoni* sporocysts in the mollusc host after primo-infection and secondary challenge. Primo-infections were performed on juvenile *B. glabrata* (5-6 mm in diameter). Snails were individually exposed for 12 h to 10 miracidia (10 Mi) in 5 ml of pond water. Ten snails were recovered 48 h after exposure and fixed in Halmi’s fixative (see below). Individual secondary challenge infections of the snails were done at 25 days after primo-infection using 10 Mi per snail. Ten snails were recovered 48 h after exposure and fixed in Halmi’s fixative (mercuric chloride 4.5%, sodium chloride 0.5%, trichloracetic acid 2%, formol 20%, acetic acid 4% and 10% of picric acid saturated water’s solution). Fixed molluscs were then dehydrated and embedded in paraffin as previously described [53,54]. Transverse histological sections, 10 µm thick, were cut and stained using azocarmine G and Heidenhain’s azan using procedures of re-hydration (toluene, 95, 70, 30% ethanol and distillate water), coloration (azocarmine G, 70% ethanol / 1% aniline, 1% acetic alcohol, distillate water, 5% Phosphotungstic acid, distillate water, Heidenhain’s azan) and dehydration (95% ethanol, absolute ethanol, toluene). Preparations were then mounted with entellan prior to microscopic examination. Pictures were taken with a Nikon MICROPHOT-FX microscope and a Nikon digital sight DS-Fi1 camera.

RNA extraction and Illumina sequencing (RNAseq)

The seven pools of 20 BgBRE snails recovered along the infection kinetic of 40 days (1DPPI [day post primo infection], 4DPPI, 15DPPI, 25DPPI, 1DPC [day post secondary challenge], 4DPC and 15DPC) were separately extracted to recover total RNA. The 2 pools of 30 naives BgBRE individuals used as control (Naive1 and Naive2) were processed in the same manner. Briefly, whole individual tissues (by pool of 10 individuals) were grinded in liquid nitrogen with pestle in mortar. The powders were re-suspended in 500 µl of Trizol reagent and stored at -80°C. Total RNA was extracted for each pool of 10 individuals according to the manufacturer’s instructions (Sigma Life Science, USA) then quality and quantity were checked using Nanodrop (Thermo Fisher Scientific). Two µg of total RNA for each sample were prepared for sequencing. Due to financial constrains, the three challenge points (i.e., 1DPC, 4DPC and 15DPC) were pooled in a single point (DPC) before sequencing.

Sequencing of the seven experimental samples (Naive1, Naive2, 1DPPI, 4DPPI, 15DPPI, 25DPPI, DPC) was performed by Montpellier GenomiX (MGX, Montpellier, France). Samples were multiplexed, 3 samples per lane using an Illumina Genome Analyzer II to generate 72 base long paired-end reads. The raw data obtained were filtered through the standard Illumina pipeline.

Transcriptome assembly

*De novo* transcriptome assembly was performed using various successive tools including, Velvet (v1.2.01), Oases (v0.2.04) and CDhit (v4.5.4). The assembly was realized without reference genome and the assembled consensus reference transcriptome were exported into a fasta file. Various parameters, including k-mer length, insert length and expected coverage, were optimized to obtain best assembly as previously described [55]. During the experimental infection the parasite is growing and developing into the mollusc host, this resulted in an increasing number of *S. mansoni* transcripts along the kinetic of infection. To solve this problem, transcripts of *S. mansoni* were deleted from the transcriptome of *B. glabrata* (transcriptome substraction). We used a Blast strategy against *Schistosoma* genome (PuertoRico sma5.2) with an identity cut-off for 77% and 60% for coverage. Finally, we simplified the transcriptome with a filtering of transcripts shorter than 198pb for which annotation results were often limited. The size of the final transcriptome was 159 711 transcripts.

Reads mapping and gene expression analysis

Before estimating genes differential expression, all the reads need to pass through a quality checking by FASTQ Parallel Groomer analysis. All the high-quality reads from each condition were aligned on the reference transcriptome assembly using the C++ script Bowtie2 (v2.0.2) running on a local devoted engine using Galaxy Project system [23]. Briefly, Bowtie2 pick up a read and try to align it end-to-end on references transcripts and attributes a score for all reads. This score is calculated by subtraction penalties for each difference between read and reference (mismatch, gap, etc) to the bonus for match. A maximum of one mismatch was allowed for the alignments and the software keep only the best alignment score by reads on the reference transcriptome. If a read has the same score for two reference transcripts, Bowtie2 make a choice by pseudo-random generator. Then, NGS tools allow transforming localization of read mapping into hits score by transcripts (Hit-Count file), required for next step. The DESeq2 software [24] (v2.12; http://www.bioconductor.org/packages/release/bioc/html/DESeq2.html) run on RStudio Software (v0.98.507 ; https://www.rstudio.com), was used for quantifying the differential gene expression. DESeq2 provides methods to test for differential expression by use of negative binomial generalized linear models; the estimates of dispersion and logarithmic fold changes (FC) incorporate data-driven prior distributions. Dispersion is calculated on the two biological duplicates of unfected snails pool (Naive1 and Naive2). An adjusted P-value cut-off of <0.1 was used to identify differentially expressed genes. Heatmaps, showing expression profiles (log2 fold change), were generated using the Cluster 3.0 software by Miyano Lab from Michael Eisen lab creation [25]. They increase view of genes regulation across the infection kinetic and Hierarchical Ascending Clustering (HAC) was chosen using Pearson correlation distance matrix with a complete linkage rule using Cluster 3.0. The created clusters were visualized with JavaTreeView (v1.1.6r4) Java (v7.51) tools.

Functional annotation

BLASTX (E-value<1e^-3^ and 20 Blast Hits to mean) searches against NCBI non-redundant nucleotide database were performed to assign a putative function to each differentially represented BgBRE transcript and ran on ABiMS Roscoff platform (France). Blast2Go Pro was used next for GO-Mapping, Annotation (E-value<1e^-6^ and CutOff 55), Interproscan (all platforms chosen), Enzyme Code & KEGG and GO-slim steps.

To demonstrate the *Biomphalaria* origin of each candidate gene, all of them that passed into annotation steps, were gone through alignment comparison stage against the *Biomphalaria* genome (genome of *Biomphalaria glabrata* Brazil available at Vectorbase web site; https://www.vectorbase.org) using Exonerate tool (v1.0.0).

**FREP RNA interference.** RNA interference of FREPs and phenotypic analysis of interfered snails RNA interference was realized using siRNA sequences that were designed to specifically knock down the expression of FREP 2, 3 and 4. Three siRNA duplexes manufactured by Eurogentec were developed to match homologous sequences in the target FREP mRNA, as follows: siRNA1(F), GUAGAUUUCUAUAGAGGCU and siRNA1(R), AGCCUCUAUAGAAAUCUAC; siRNA2(F), CGGCUUUGGAGAUUACAAC and siRNA2(R), GUUGUAAUCUCCAAAGCCG; and siRNA3(F), CCGCUUUAAUUGAAGAGAU and siRNA3(R), AUCUCUUCAAUUAAAGCGG. To improve the efficiency of siRNA delivery, we used Invivofectamine (Invitrogen) according to the manufacturer’s recommendations. Briefly, 18 μg of siRNA duplexes were diluted in 6 μl of complexation buffer, mixed with 12 μl of Invivofectamine, and then incubated for 30 min at 50°C. After this incubation, 400 μl PBS-snail was added to the mixture and dialysis was performed for 2 hours in 1 L of room-temperature PBS-snail, using a FloatALyzer G2 cassette (Spectrum Medical Laboratories). Then, 200 ng of pooled duplex siRNA was injected into the cardiac sinus of each *B. glabrata* snail, as previously described [26]. As a control, the above protocol was repeated using siRNA targeting a GFP reporter gene (commercial GFP siRNA were used; Eurogentec). The efficiency of FREP knockdown was determined by Q-RT-PCR performed on total RNA recovered from groups of 10 snails assessed at 4 days post injection of FREP siRNA, GFP siRNA, as well as un-injected snails.

The resulting knockdown snails were then assessed for their level of protection against secondary infection. Ninety *B. glabrata* BRE snails (8-9 mm in diameter) were individually exposed to 10 miracidia (10 Mi) of SmBRE in 5 ml of pond water. At 21 days after the primo- infection, 30 snails were individually injected with FREP or GFP siRNA, and the remaining 30 snails were kept untreated (control). At 25 days after the primo-infection, the snails were subjected to secondary challenge with 10 Mi SmBRE. At the same time, 30 BgBRE snails were exposed to 10 Mi SmBRE and used as a control of the secondary challenge infection. Fifteen days later, all snails were fixed and exhaustively dissected, and the presence of SpIs in snail tissue was used to determine the percentage of infected snails (prevalence).

**Proteomics electrophoresis and LC-MS/MS Analysis**

Snail plasma was recovered along a kinetic of innate immune memory. BgBRE snails were infected with 10 miracidia of SmBRE and 25 days after the primo infection the snails were secondary challenged with 10 miracidia of SmBRE (Figure 1). For all the experiments the success of the primo-infection was attested by the presence of SpII in the hepatopancreas and only snails harbouring SpII were individually secondary challenged. Fifteen days post challenge (15 DPC) to confirm the success of the innate immune memory process, snails were fixed (in Raillet-Henry's solution) and exhaustively dissected, and the presence of SpIs in snail tissue was used to determine the percentage of infected snails (prevalence).. Because of the difference in the age (and size) between the SpI from the primo-infection (40 days old at fixation time) and those from the challenge (15 days old at fixation time), it was easy to distinguish them.

Along this kinetic of infection four plasma samples were collected: (i), a control sample recovered from naïve snails (before the primo-infection); (ii), a 15 Day Post Primo Infection (DPPI) sample recovered after SpI development and spII asexual multiplication; (iii), a 25DPPI sample recovered after the spII migration and before the secondary challenge and (iv), a 15 days post secondary challenge (15 DPC) sample (Fig. 1). For each sample, the hemolymph was collected from the head-foot region of 50 BgBRE snails as previously described [44,56,57]. Hemocytes were removed by centrifugation (2 500 rpm; 10 min; 4°C) and the plasma was recovered. Then, hemoglobin was removed from plasma using an ultra-centrifugation (55 000 rpm; 2.5 hours; 4°C) (Fig. 1). Quantification of total protein concentration was performed with the 2D Quant Kit and plasmas were storaged at -80°C until use.

Proteomic comparative approach was conducted by 2D gel electrophoresis on the four plasma samples previously described. For each sample (Naïve, 15DPPI, 25DPPI and 15DPC) 100 µg of plasma were lyophilised and resuspended in 2D lysis buffer (8 M urea, 40 mM Tris, 4% CHAPS, 65 mM DTT) to solubilise and denaturate the proteins. The ﬁrst dimension of 2D gel was performed on 17 cm Ready IPG Strips, pH 3–10 non-linear gradient (Bio-Rad). Isoelectrofocusing (IEF) was performed as previously described with a gradual increasing voltage to 8000 V and running for 90,000 Vh at 20°C[54, 58]. Second dimension SDS-PAGE electrophoresis was performed in 12% acrylamide gels and proteins were visualized by silver staining. 2D gels were scanned using a densitometer (GS-800 Calibrated Densitometer, Bio-Rad). Although the silver stain is the most sensitive currently available, the protocol can generate staining differences within and between gels, notably in the case of background staining. Thus to avoid spurious differences, gels with similar spot intensities and background coloration were selected using an analytical approach [54]. Five gels for each experimental sample were selected to perform the comparative proteomic analysis. Replicate proteome images for each condition were aligned and matched to generate a composite map image. Comparative analysis of digitized proteome maps was performed using the image analysis software PDQuest 7.4.0 (Bio-Rad). Signiﬁcant differences in protein spot patterns and/or intensities (at least 2 fold) were identiﬁed by pair-wise comparisons of spots between gel images in naïve versus 15DPPI; 15DPPI versus 25DPPI and 25DPPI versus 15DPC proteomes.

Gel plugs containing the proteins of interest were excised from the gel that was silver-stained using a method compatible with mass spectrometry. Plugs were digested with trypsin and analysed using a nanoscale capillary liquid chromatography Ultimate 3000 coupled to a LTQ-Orbitrap tandem mass spectrometer (nanoLC–MS/MS).

The resulting peptide MS/MS spectra were processed and converted into peak lists in dta format using the SEQUEST algorithm for interrogation of protein or nucleotide sequence databases (Swiss prot-trembl, *Schistosoma mansoni* Express sequence tag and *Biomphalaria glabrata* Brazil transcriptome (available on IHPE website bioinformatics resources (<http://2ei.univ-perp.fr/?page_id=89>)) using Mascot (http://www.matrixscience.com/). No missed cleavages were allowed and some variable modifications were taken into account in the search such as acetylation (protein N-term), oxidation and dioxidation (M), trioxidation (C). Searches were performed using an error on experimental peptide mass values of ± 15.0 ppm and an error for MS/MS fragment ion mass values of 1.0 Da.

Mascot results were validated using IRMa software (interpretation of Mascot results) developed by "EDyP Service" laboratory (Grenoble, France). IRMa avoids redundant proteins in the analysis and reduced false positive to less than 1%. A protein is considered to be correctly identified if at least two peptides were confidently matched with database sequences. In addition an overall Mascot score was given for each peptide, a score greater than 50 was considered significant (*p* < 0.05) [29].

**Plasma transfer**

To investigate the implication of plasmatic factors in innate immune memory, an experiment of plasma transfer was conducted. Hemolymph from 15 days infected snails was recovered and hemocytes were removed by centrifugation (5 min at 2500 rpm). Hemolymph from naïve snails was recovered following the same procedure and was used as control. Plasmas were frozen at -80°C until used. Twenty five naive snails were injected with 20 µL of plasma from infected snails and twenty three with plasma from naïve snails. We also injected saline solution (TBS tween : 150 mM of Tris, 20 mM of NaCl and 0.05% Tween 20) to 25 snails as a control of injection. Fifteen days after injections, all the experimental groups were infected with 10 Mi of SmBRE and 48 naïve snails were also exposed to 10 Mi and were used as control of the infection. Fifteen days after infections, snails were fixed and the primary sporocyst (SpI) numbers they harboured were determined following previously described methods [27,28]. Briefly, snails were relaxed in pond water containing excess crystaline menthol for 6 h. The snail body was removed from the shell and fixed in modified Raillet-Henry's solution [59]. Using this technique, SpI were readily observable as translucent white bodies within an opaque yellow tissue background. The number of SpI present in each snail was determined following exhaustive dissection to determine the prevalence of infection.

### Quantitative Real time PCR (Q-RT-PCR)

Q-RT-PCR analyses were conducted to analyze the expression of randomly selected target in Naive1/Naive2 condition compare to RNAseq experiment.

Reverse transcription was performed on the same sample than RNAseq experiment and according to previously described procedures [60]. cDNA were kept at -80°C until used for Q-RT-PCR analysis. Q-RT-PCR analyses were performed using a LightCycler 2.0 system (Roche Applied Science) and a Light Cycler Fast start DNA Master SYBR Green I kit (Roche Applied Science). Q-RT-PCR reactions were set up according to the Light Cycler Manual (Roche Molecular Biochemials, Germany) as previously described [60]. Briefly, Q-RT-PCR amplification was performed using 2.5 µl of cDNA in a final volume of 10 µl containing 3 m M MgCl_2_, 0.5 µM of each primer and 1 µl of master mix. Relevant candidate genes identified in the proteomic approach were selected and specific primers were designed using either the LightCycler probe design software version 1.0 (Roche) or the PerlPrimer software and are given in Supplementary primers. The following Light-Cycler run protocol was used: denaturation at 95°C for 10 min, followed by 40 cycles of amplification and quantification at 95°C for 10 s, 60°C for 5 s and 72°C for 16 s, a melting curve of 60-95°C with a heating rate of 0.1°C/s and continuous fluorescence measurement, and then a cooling step to 40°C. For each reaction, the cycle treshold (Ct) was determined using the “Fit Point Method” of the LightCycler Software, version 3.3 (Roche Diagnostics). The PCR reactions were performed in triplicate and the mean Ct values were calculated. For each mRNA to be analysed, the absence of contaminating genomic DNA was verified by running a no-RT control using primers for the S19 RNA.

**Statistical analysis**

To compare prevalences between all the conditions a Fischer’s exact test was used. The difference was considered significant when the P value of the test was under 0.05.

For transcriptomic approach, significant values are considered below 0.1 thanks to DESeq2 calculation. For the proteomic approach statistical analysis associated with quantitative differences between spots was performed using a Mann-Whitney U test directly available in PDQuest 7.4.0 software (Bio-Rad).

**References**

1. Loker ES, Adema CM, Zhang SM, Kepler TB (2004) Invertebrate immune systems--not homogeneous, not simple, not well understood. Immunol Rev 198: 10-24.

2. Kurtz J (2004) Memory in the innate and adaptive immune systems. Microbes Infect 6: 1410-1417.

3. Kurtz J, Franz K (2003) Innate defence: evidence for memory in invertebrate immunity. Nature 425: 37-38.

4. Portela J, Duval D, Rognon A, Galinier R, Boissier J, et al. (2013) Evidence for specific genotype-dependent immune priming in the lophotrochozoan Biomphalaria glabrata snail. J Innate Immun 5: 261-276.

5. Rodrigues J, Brayner FA, Alves LC, Dixit R, Barillas-Mury C (2010) Hemocyte differentiation mediates innate immune memory in Anopheles gambiae mosquitoes. Science 329: 1353-1355.

6. Sadd BM, Schmid-Hempel P (2006) Insect immunity shows specificity in protection upon secondary pathogen exposure. Curr Biol 16: 1206-1210.

7. Lavine MD, Strand MR (2002) Insect hemocytes and their role in immunity. Insect Biochem Mol Biol 32: 1295-1309.

8. Zanker KS (2010) Immunology of Invertebrates: Humoral. Encyclopedia of Life Sciences John Wiley & Sons, Ltd: Chichester.

9. Netea MG, Quintin J, van der Meer JW (2011) Trained immunity: a memory for innate host defense. Cell Host Microbe 9: 355-361.

10. Rowley AF, Powell A (2007) Invertebrate immune systems specific, quasi-specific, or nonspecific? J Immunol 179: 7209-7214.

11. Wang J, Wang L, Yang C, Jiang Q, Zhang H, et al. (2013) The response of mRNA expression upon secondary challenge with Vibrio anguillarum suggests the involvement of C-lectins in the immune priming of scallop Chlamys farreri. Dev Comp Immunol 40: 142-147.

12. Roth O, Kurtz J (2009) Phagocytosis mediates specificity in the immune defence of an invertebrate, the woodlouse Porcellio scaber (Crustacea: Isopoda). Dev Comp Immunol 33: 1151-1155.

13. Pope EC, Powell A, Roberts EC, Shields RJ, Wardle R, et al. (2011) Enhanced cellular immunity in shrimp (Litopenaeus vannamei) after 'vaccination'. PLoS One 6: e20960.

14. Hauton C, Smith VJ (2007) Adaptive immunity in invertebrates: a straw house without a mechanistic foundation. Bioessays 29: 1138-1146.

15. Little TJ, Colegrave N, Sadd BM, Schmid-Hempel P (2008) Studying immunity at the whole organism level. Bioessays 30: 404-405; author reply 406.

16. Sire C, Rognon A, Théron A (1998) Failure of *Schistosoma mansoni* to reinfect *Biomphalaria glabrata* snails : acquired humoral resistance or intra-specific larval antagonism ? Parasitology 117: 117-122.

17. Adema CM, Bayne CJ, Bridger JM, Knight M, Loker ES, et al. (2012) Will all scientists working on snails and the diseases they transmit please stand up? PLoS Negl Trop Dis 6: e1835.

18. Sanchez Alvarado A (2013) On the trail of a tropical disease. Elife 2: e01115.

19. Theron A, Pages JR, Rognon A (1997) Schistosoma mansoni: Distribution patterns of miracidia among Biomphalaria glabrata snail as related to host susceptibility and sporocyst regulatory processes. Experimental Parasitology 85: 1-9.

20. Roger E, Mitta G, Mone Y, Bouchut A, Rognon A, et al. (2008) Molecular determinants of compatibility polymorphism in the Biomphalaria glabrata/Schistosoma mansoni model: New candidates identified by a global comparative proteomics approach. Molecular and Biochemical Parasitology 157: 205-216.

21. Gourbal BEF, Gabrion C (2006) Histomorphological study of the preputial and clitoral glands in BALB/c mice with experimental Taenia crassiceps infections. Journal of Parasitology 92: 189-192.

22. Dheilly NM, Duval D, Mouahid G, Emans R, Allienne JF, et al. (2015) A family of variable immunoglobulin and lectin domain containing molecules in the snail Biomphalaria glabrata. Dev Comp Immunol 48: 234-243.

23. Giardine B, Riemer C, Hardison RC, Burhans R, Elnitski L, et al. (2005) Galaxy: a platform for interactive large-scale genome analysis. Genome Res 15: 1451-1455.

24. Love MI, Huber W, Anders S (2014) Moderated estimation of fold change and dispersion for RNA-seq data with DESeq2. Genome Biol 15: 550.

25. Eisen MB, Spellman PT, Brown PO, Botstein D (1998) Cluster analysis and display of genome-wide expression patterns. Proc Natl Acad Sci U S A 95: 14863-14868.

26. Baeza Garcia A, Pierce RJ, Gourbal B, Werkmeister E, Colinet D, et al. (2010) Involvement of the cytokine MIF in the snail host immune response to the parasite Schistosoma mansoni. PLoS Pathog 6.

27. Allienne JF, Theron A, Gourbal B (2011) Recovery of primary sporocysts in vivo in the Schistosoma mansoni/Biomphalaria glabrata model using a simple fixation method suitable for extraction of genomic DNA and RNA. Exp Parasitol 129: 11-16.

28. Moné Y, Mitta G, Duval D, Gourbal BE (2010) Effect of amphotericin B on the infection success of *Schistosoma mansoni* in *Biomphalaria glabrata*. Exp Parasitol 125: 70-75.

29. Beltran S, Gourbal B, Boissier J, Duval D, Kieffer-Jaquinod S, et al. (2010) Vertebrate host protective immunity drives genetic diversity and antigenic polymorphism in Schistosoma mansoni. Journal of Evolutionary Biology 24: 554-572.

30. Damian RT (1997) Parasite immune evasion and exploitation: reflections and projections. Parasitology 115 Suppl: S169-175.

31. Guillou F, Roger E, Mone Y, Rognon A, Grunau C, et al. (2007) Excretory-secretory proteome of larval Schistosoma mansoni and Echinostoma caproni, two parasites of Biomphalaria glabrata. Mol Biochem Parasitol 155: 45-56.

32. Zhang SM, Nian H, Wang B, Loker ES, Adema CM (2009) Schistosomin from the snail Biomphalaria glabrata: expression studies suggest no involvement in trematode-mediated castration. Mol Biochem Parasitol 165: 79-86.

33. Davies SJ, Chapman T (2006) Identification of genes expressed in the accessory glands of male Mediterranean Fruit Flies (Ceratitis capitata). Insect Biochem Mol Biol 36: 846-856.

34. Ram KR, Wolfner MF (2009) A network of interactions among seminal proteins underlies the long-term postmating response in Drosophila. Proc Natl Acad Sci U S A 106: 15384-15389.

35. Koene JM, Sloot W, Montagne-Wajer K, Cummins SF, Degnan BM, et al. (2010) Male accessory gland protein reduces egg laying in a simultaneous hermaphrodite. PLoS One 5: e10117.

36. Kurtz J (2005) Specific memory within innate immune systems. Trends Immunol 26: 186-192.

37. Vazquez L, Alpuche J, Maldonado G, Agundis C, Pereyra-Morales A, et al. (2009) Review: Immunity mechanisms in crustaceans. Innate Immun 15: 179-188.

38. Cooper EL, Rinkevich B, Uhlenbruck G, Valembois P (1992) Invertebrate immunity: another viewpoint. Scand J Immunol 35: 247-266.

39. Little TJ, Kraaijeveld AR (2004) Ecological and evolutionary implications of immunological priming in invertebrates. Trends Ecol Evol 19: 58-60.

40. Zhang SM, Adema CM, Kepler TB, Loker ES (2004) Diversification of Ig superfamily genes in an invertebrate. Science 305: 251-254.

41. Zhang SM, Loker ES (2003) The FREP gene family in the snail Biomphalaria glabrata: additional members, and evidence consistent with alternative splicing and FREP retrosequences. Fibrinogen-related proteins. Developmental and Comparative Immunology 27: 175-187.

42. Adema CM, Hertel LA, Miller RD, Loker ES (1997) A family of fibrinogen-related proteins that precipitates parasite-derived molecules is produced by an invertebrate after infection. Proc Natl Acad Sci U S A 94: 8691-8696.

43. Hanington PC, Lun CM, Adema CM, Loker ES (2010) Time series analysis of the transcriptional responses of Biomphalaria glabrata throughout the course of intramolluscan development of Schistosoma mansoni and Echinostoma paraensei. Int J Parasitol 40: 819-831.

44. Moné Y, Gourbal B, Duval D, Du Pasquier L, Kieffer-Jaquinod S, et al. (2010) A Large Repertoire of Parasite Epitopes Matched by a Large Repertoire of Host Immune Receptors in an Invertebrate Host/Parasite Model. PLoS Negl Trop Dis 4: e813.

45. Hanington PC, Forys MA, Dragoo JW, Zhang SM, Adema CM, et al. (2010) Role for a somatically diversified lectin in resistance of an invertebrate to parasite infection. Proc Natl Acad Sci U S A 107: 21087-21092.

46. Dheilly NM, Nair SV, Smith LC, Raftos DA (2009) Highly variable immune-response proteins (185/333) from the sea urchin, Strongylocentrotus purpuratus: proteomic analysis identifies diversity within and between individuals. J Immunol 182: 2203-2212.

47. Galinier R, Portela J, Mone Y, Allienne JF, Henri H, et al. (2013) Biomphalysin, a New beta Pore-forming Toxin Involved in Biomphalaria glabrata Immune Defense against Schistosoma mansoni. PLoS Pathog 9: e1003216.

48. Endo Y, Mitsui K, Motizuki M, Tsurugi K (1987) The mechanism of action of ricin and related toxic lectins on eukaryotic ribosomes. The site and the characteristics of the modification in 28 S ribosomal RNA caused by the toxins. J Biol Chem 262: 5908-5912.

49. Chumkhunthod P, Rodtong S, Lambert SJ, Fordham-Skelton AP, Rizkallah PJ, et al. (2006) Purification and characterization of an N-acetyl-D-galactosamine-specific lectin from the edible mushroom Schizophyllum commune. Biochim Biophys Acta 1760: 326-332.

50. Oda T, Tsuru M, Hatakeyama T, Nagatomo H, Muramatsu T, et al. (1997) Temperature- and pH-dependent cytotoxic effect of the hemolytic lectin CEL-III from the marine invertebrate Cucumaria echinata on various cell lines. J Biochem 121: 560-567.

51. Pereira CA, Martins-Souza RL, Correa A, Jr., Coelho PM, Negrao-Correa D (2008) Participation of cell-free haemolymph of Biomphalaria tenagophila in the defence mechanism against Schistosoma mansoni sporocysts. Parasite Immunol 30: 610-619.

52. Mattos AC, Kusel JR, Pimenta PF, Coelho PM (2006) Activity of praziquantel on in vitro transformed Schistosoma mansoni sporocysts. Mem Inst Oswaldo Cruz 101 Suppl 1: 283-287.

53. Gourbal BE, Gabrion C (2006) Histomorphological study of the preputial and clitoral glands in BALB/c mice with experimental Taenia crassiceps infections. J Parasitol 92: 189-192.

54. Roger E, Mitta G, Moné Y, Bouchut A, Rognon A, et al. (2008) Molecular determinants of compatibility polymorphism in the *Biomphalaria glabrata/Schistosoma mansoni* model: New candidates identified by a global comparative proteomics approach. Molecular and Biochemical Parasitology 157: 205-216.

55. Dheilly NM, Adema C, Raftos DA, Gourbal B, Grunau C, et al. (2014) No more non-model species: the promise of next generation sequencing for comparative immunology. Dev Comp Immunol 45: 56-66.

56. Bouchut A, Coustau C, Gourbal B, Mitta G (2007) Compatibility in the Biomphalaria glabrata/Echinostoma caproni model: new candidate genes evidenced by a suppressive subtractive hybridization approach. Parasitology 134: 575-588.

57. Bouchut A, Coustau C, Gourbal B, Mitta G (2007) Compatibility in the Biomphalaria glabrata/Echinostoma caproni model: new candidate genes evidenced by a suppressive subtractive hybridization approach. Parasitology 134: 575-588.

58. Beltran S, Gourbal B, Boissier J, Duval D, Kieffer-Jaquinod S, et al. (2011) Vertebrate host protective immunity drives genetic diversity and antigenic polymorphism in Schistosoma mansoni. J Evol Biol 24: 554-572.

59. Pointier JP, Paraense WL, Dejong RJ, Loker ES, Bargues MD, et al. (2002) A potential snail host of schistosomiasis in Bolivia: Biomphalaria amazonica Paraense, 1966. Mem Inst Oswaldo Cruz 97: 793-796.

60. Guillou F, Mitta G, Dissous C, Pierce R, Coustau C (2004) Use of individual polymorphism to validate potential functional markers: case of a candidate lectin (BgSel) differentially expressed in susceptible and resistant strains of Biomphalaria glabrata. Comp Biochem Physiol B Biochem Mol Biol 138: 175-181.
