# Supplementary material for: A Shift from Cellular to Humoral Responses Contributes to Innate Immune Memory in the Vector Snail Biomphalaria glabrata
Source: PLoS Pathog. 2016 Jan 6;12(1):e1005361. doi: 10.1371/journal.ppat.1005361 (PMC4703209; doi:10.1371/journal.ppat.1005361)
Supplement: S2 Appendix — (DOCX) [file ppat.1005361.s006.docx]

**Appendix S2: Trade-off between reproduction and immunity.**

After infection of *B. glabrata* with *S. mansoni*, a parasitic castration occurs, taking full effect by 25 to 30 days post-infection [61-63]. The maintenance of efficient innate immune memory response for the snail’s entire lifespan would result in a huge energetic loss, presumably resulting in trade-offs with other life-history traits [64,65]. Interestingly, we herein observed down-regulation of molecules involved in gametogenesis following primary infection and secondary challenge. This provides molecular-level confirmation of the parasitic castration previously observed by many authors, and provides evidence for the existence of a potential trade-off between reproduction and the maintenance of an efficient innate immune memory response in *B. glabrata* snails.

**References**

61. Theron A, Gerard C, Mone H (1992) Early enhanced growth of the digestive gland of Biomphalaria glabrata infected with Schistosoma mansoni: side effect or parasite manipulation? Parasitol Res 78: 445-450.

62. Theron A, Mone H, Gerard C (1992) Spatial and energy compromise between host and parasite: the Biomphalaria glabrata-Schistosoma mansoni system. Int J Parasitol 22: 91-94.

63. Faro MJ, Perazzini M, Correa Ldos R, Mello-Silva CC, Pinheiro J, et al. (2013) Biological, biochemical and histopathological features related to parasitic castration of Biomphalaria glabrata infected by Schistosoma mansoni. Exp Parasitol 134: 228-234.

64. Hangartner S, Sbilordo SH, Michalczyk L, Gage MJ, Martin OY (2013) Are there genetic trade-offs between immune and reproductive investments in Tribolium castaneum? Infect Genet Evol 19C: 45-50.

65. McNamara KB, Wedell N, Simmons LW (2013) Experimental evolution reveals trade-offs between mating and immunity. Biol Lett 9: 20130262.
